# Examining the spiral of silence in offline and online expression of public opinion on recruitment of women into the Kuwaiti military

**DOI:** 10.3389/fsoc.2025.1558439

**Published:** 2025-05-02

**Authors:** Ali Alkandari, Edward Frederick, Ahmad R. Alsaber, Fatimah Alhashem

**Affiliations:** ^1^Mass Communication Department, Gulf University for Science and Technology, Hawally, Kuwait; ^2^Greenhill Center of the Arts, University of Wisconsin-Whitewater, Whitewater, WI, United States; ^3^College of Business and Economics, American University of Kuwait, Safat, Kuwait; ^4^Ministry of Education, Kuwait City, Kuwait

**Keywords:** spiral of silence, religiosity, opinion expression, online media, Kuwaiti military

## Abstract

This study used the Spiral of Silence (SOS) and Uses and Gratifications (U&G) framework to examine the influence of religion on offline and online expressions of opinion about women in the Kuwaiti military. A self-administered questionnaire was completed by 652 university students in Kuwait. Data were analyzed using Multiple Linear Regression (MLR) to identify predictors of opinion expression in offline and online settings. Predictor variables included fear of social isolation, internally and externally driven religious fears, religiosity, and media use behaviors. Our results revealed culturally oriented factors, including fear of social isolation and externally driven religious fear, predicted avoidance of expressing opinions in offline contexts. On the other hand, personality-oriented factors, including personal religious inclinations, “social use of religious media,” and frequency of occasions spent using the X platform, triggered the expression of opinion on the topic in the online context. The results are discussed in terms of the influence of online media in an Arab and Muslim culture. The influence of online media can have a liberating effect in a “solid” and “static” religious public opinion climate. This study suggests that when online discussions regarding women issues are circulated offline by people, the discussions can have transformative effects on the restrictive culture.

## 1 Introduction

Recent cultural changes worldwide demonstrate that a study relating public opinion to religion is relevant, even in Western societies. Religion can no longer be confined to the private sphere or roles serving personal and subjective needs (Mosher, 1989). In an increasingly cosmopolitan world, where immigrants pour into Western nations and start contributing to politics, isolating religion from public life is no longer possible. Chaudhry and Gruzd (2020) noticed an increased use of religion and religious rhetoric by people commenting on social media about various social issues. Eckstein and Turman (2002) argued that Western education needs to stop trying to suppress religious voices because religions provide the moral and ethical frame of reference for many people's judgments about social dilemmas. Carter (1993) argued, “The religions, for all their arrogance and sinfulness, can often provide approaches to the considerations of ultimate questions that a world yet steeped in materialistic ideologies desperately require” (p. 273).

Open criticism of religion in the arenas of public opinion can be life-threatening, especially in many Middle Eastern nations. Criticism or ridicule of Islam can lead to violent crime (Ahmad and Younas, 2021). Ahmad and Younas (2021) stated, “People are not only openly condemned, threatened but are also killed for voicing their opinions when it comes to highly controversial and sensitive religious issues” (p. 212). Among the Pashtun people of Pakistan, young people silenced their negative opinions about Islam on social media for fear of isolation and physical harm (Ahmad and Younas, 2021). The centrality of Islam to public opinion in the Middle East is evident in the many cases where religious communities provide opinions on leadership and mobilize people around certain matters of public concern. Such cases are related to violent demonstrations against satirical cartoons of the Prophet Muhamad and the burning of the Holy Koran in Europe (Chaudhry and Gruzd, 2020; Fladmoe and Steen-Johnsen, 2017).

In Kuwait, a Middle Eastern country, the announcement by the Minister of Defense that women were to be recruited for military service is a case that illustrates the central role of Islam in public opinion. The Minister's announcement triggered an intense public debate and backlash from the religious community. Conservative members of parliament initiated a parliamentary inquiry into the Minister's reasons for the policy change. To avoid a vote of no confidence, the minister issued a statement pointing out that the recruitment of women would follow the dictates of a religious edict (fatwa) issued by the Kuwaiti Council for Fatwa and Legal Legislation. The fatwa mandated that female recruits be separated from male recruits in military camps, that women be assigned only to medical and ancillary units, and that they be excluded from military drills and tasks involving weapons and other military equipment. In addition, the ministry stated that it would require the consent of the direct male guardians of female recruits. Many women's rights activists argued that these restrictions were backward and violated the country's constitutional articles on equal rights (Human Rights Watch, 2023). The minister's capitulation is an example of the continuing influence of religion on public opinion in Kuwait.

The current study relates religion to public opinion. The spiral of silence (SOS) theory proposed by Noelle-Neumann (1977) serves as the theoretical framework of this study because this theory explores issues of opinion expression and public opinion, the main interest of this study. The SOS theory examines the formation of public opinion and its influence on the likelihood that individuals will speak out on controversial social issues. In her seminal work, Noelle-Neumann (1977) argues that social control influences individuals, particularly those who see themselves as representing a minority view or opinion on an issue. This group tends to remain silent about their opinion because they fear social isolation (FSI) and the social sanctions the majority may impose to maintain social consensus.

By relating religion to SOS, this study contributes to the literature in two ways. First, it responds to calls from SOS scholars for “a more macroscopic focus” (Scheufele and Moy, 2000, p. 3) where determining “cultural and social conditions in a society” (Donsbach and Stevenson, 1984, p. 15; El-Dabt et al., 2025) influence the silencing of opinions in public debates. Western perspectives on SOS generally emphasize the role of people's personality and psychology over culture (Croucher et al., 2014). Second, the study follows a call by Noelle-Neumann (1991) to associate SOS with religion; “In theology and philosophy, its (SOS's) application might be in moral philosophy, where actions arising from humans' social nature are subject to a new assessment” (Noelle-Neumann, 1991, p. 282). However, Noelle-Neumann made this claim as early as 1991; only a few studies link SOS to religion (Alkazemi, 2015). Only a few scholars (e.g., Alkazemi, 2013; Croucher et al., 2014) have attempted to examine this area.

This study addresses a controversial issue in public opinion in Kuwait, the recruitment of women into the military, by examining the influence of four sets of predictors on the likelihood of people expressing an opinion offline and online. The first predictors include religious predispositions (social and personal religious beliefs and religious rituals). The second consists of the theoretical components of SOS (FSI and the congruence of personal opinion with that of the public). The third set relates to media usage (frequency of exposure to religious media content, use of X's media platform, and the “individual” and “social” Uses and Gratifications (U&G) reasons for using religious media content). The fourth group of predictors is the concepts of internally and externally driven religious fears that influence the likelihood of expressing opinions. Since every society and culture consensually institutionalizes the views and behaviors of its people (Scheufele and Moy, 2000), it is not sufficient to examine the social control mechanisms of SOS only in the West.

### 1.1 A religious contextualization of women recruitment in the military

The social setting of this study is the state of Kuwait, where Islam is the most salient cultural predictor of attitudes and beliefs (Al-Kandari et al., 2022). Of the 5 million people living in Kuwait, all are Muslims, except for about 300 Christians. The Kuwaiti economy is mainly driven by oil and is a constitutional monarchy (CIA Factbook, 2022).

Several articles of the Kuwaiti constitution emphasize respect for Islamic jurisprudence in social and legal matters. Article 2 states, “The religion of the State is Islam, and the Islamic Shari'a shall be a main source of legislation.” The constitution also states that the government protects religious freedom as long as it is “in accordance with established customs and does not conflict with public policy or morals” (Kuwaiti Constitution, 1962). In many situations, the government uses the “conflict” clause to restrict religious freedoms by arguing that Kuwait must “protect the heritage of Islam.” Although the government generally does not interfere with religious practices, there are reports that it delays and/or prevents the construction of houses of worship for the Kuwaiti Shia minority and expatriate members of other religions (U.S. Department of State, 2021).

Women were granted the right to vote in 2005. Before that, Kuwait's Islamist members of parliament had used conservative religious interpretations to prevent women's political participation. “The Islamists continued to argue that a woman's primary and divinely ordained role was to be a wife and mother, and was forbidden by religious teachings to become involved in politics” (Meyer et al., 2007, p. 294). In 1985, political Islamists succeeded in rejecting a bill granting women the right to vote by using a fatwa from the Ministry of Islamic Endowments and Islamic Affairs, which stated that “The nature of the electoral process befits men, who are endowed with ability and expertise; it is not permissible for women to recommend or nominate other women or men” (Olimat, 2009). This persisted throughout the 1990s; “(W)omen's rights would decrease due to the backlash coming from the increasingly powerful Islamist/tribalist blocs in parliament who used religion and tradition as justifications for their resistance” (Meyer et al., 2007, p. 295).

The controversy over the recruitment of women by the Kuwaiti military has divided public opinion since the Minister of Defense first announced the plan in 2017. The debate intensified in 2021 when the new defense minister stated that enlisting the first group of women was planned for the future (Human Rights Watch, 2023). The conservative religious community submitted fatwas arguing that the move was against Islam because it encouraged women to mingle with men, that it unnecessarily violated the divine order of gender roles, and that it was unnecessary as long as there were ample unemployed men suitable for conscription to perform military duties (Abdulgafar, 2018). Other clerics argued that women were not physically suited to strenuous military duties and that service in the military's ancillary, administrative, healthcare, and medical branches was more suitable (Alasidan, 2018). However, women's rights activists argued that military recruitment was comparable to women joining the police force, a necessary and constitutional step toward gender equality (Alkhaldi, 2021).

### 1.2 Spiral of silence (SOS)

SOS is a theory that focuses on individuals' fear of being isolated if they express minority opinions and on the effects of self-censorship on public opinion (Noelle-Neumann, 1977, 1991, 1993; Noelle-Neumann and Petersen, 2004). SOS argues that the spiral begins when a commonly held opinion on an issue dominates public discourse. Much of the SOS research focuses, in particular, on discourse in the news media. As an opinion becomes more prevalent, the audience begins to perceive this opinion as that of the majority. Those who hold different views begin to sense that they are in the minority and start to remain silent for fear of being isolated. As a result, the supposed majority opinion dominates the public discourse even more, encouraging those who see themselves in the minority to remain even more silent. What follows is a spiral in which the perceived majority opinion appears more and more frequently in public discourse, and other opinions appear less and less often. This spiral progresses gradually as the majority opinion occupies more space in the news media and other arenas of public discourse (Scheufele and Moy, 2000). Noelle-Neumann explains that the silencing effect of fear of isolation will occur for all but a few “avant-garde” or “hardcore” individuals who will express their accurate opinions even in the face of possible isolation (1993, 2004).

According to SOS scholars, individuals look for clues about popular opinions in public discourse (Donsbach and Stevenson, 1984, p. 7). The news media is a particularly important source of clues about public opinion. People whose opinions do not match these cues remain silent and do not express their views. The views presented in the media and other forms of popular culture do not have to be the actual majority opinion for the cues to have a silencing effect as long as the audience perceives them as the majority (Donsbach and Stevenson, 1984). In contrast, people will express their opinions more freely if they find that they are congruent with the majority. When the media reports positively and frequently on an issue, people tend to believe that the issue enjoys broader public support and are, therefore, more likely to express their opinions freely, while negative comments in the media have the opposite effect (Noelle-Neumann, 1977, 1991, 1993).

### 1.3 Research questions and hypotheses

#### 1.3.1 Religiosity and opinion expression

The few available SOS studies that link SOS to religion indicate that religiosity correlates with the likelihood of expressing an opinion. Croucher et al. (2014) found that individuals in the United States who reported higher levels of religiosity were more likely to express an opinion on an election, even in socially incongruent opinion climates. Ho et al. (2013) found that individuals who strongly believed religion would guide them in life were more willing to justify their opinions. Alkazemi (2013) also found that religiosity correlated positively with willingness to communicate about religious topics.

Kuwaitis often reported higher levels of religiosity than Americans (Abdul-Khalek and Lester, 2003; Abdel-Khalek and Lester, 2012), which supports the suggestion that commitment to religion strongly influences the expression of opinions by Kuwaitis. Recent SOS studies in Kuwait examining the expression of the views on gender segregation in public schools (Al-Sumait et al., 2021) and the inclusion of women in the police force (Al-Kandari et al., 2022) suggest that Kuwaitis are more or less likely to express an opinion on a particular policy depending on their religious stance. These studies generally argue that individuals committed to religion feel obligated to defend Islam or cover the problem with a religious point of view (Al-Kandari et al., 2022; Al-Sumait et al., 2021; Dashti et al., 2015). The studies also argue that religious dogmas make people feel morally right while others are wrong. These are the “avant-garde” or “hardcore” that Noelle-Neumann (1993); Noelle-Neumann and Petersen (2004) identified, the outspoken people who are willing to defend their religious views even if they are in the minority. As a result, the following hypothesis is posited:

H1: Religiously committed individuals are more likely to express their opinions about Kuwait's recruitment of women into the military.

#### 1.3.2 FSI and opinion expression

According to SOS, the fear of social isolation (FSI) contributes to the stifling of opinions (Noelle-Neumann, 1977, 1991). People look for cues about the prevailing views in their environment to assist them in deciding whether to speak out because “(t)he effort spent in observing the environment is apparently a smaller price to pay than the risk of losing the goodwill of one's fellow human beings, of becoming rejected, despised, alone” (Noelle-Neumann, 1993, p. 41). A majority imposes social control to maintain social cohesion, which “must be constantly ensured by a sufficient level of agreement on values and goals” (Noelle-Neumann, 1991, p. 158).

Cultural repression and people's history of negative interactions (Neuwirth et al., 2007) contribute to avoiding expressing opinions. SOS studies indicate that the FSI negatively predicts opinion expression (e.g., Burnett et al., 2022; Chaudhry and Gruzd, 2020).

At the Kuwaiti cultural group level, there have been several cases of Kuwaitis being coerced by family members and society in general to convert from Islam to other religions. In January 2021, a well-known TV journalist and presenter posted a video on Snapchat declaring his conversion to Christianity. Reactions to his conversion on social media were mixed. While some supported his freedom to think and choose, others publicly prayed for his return to the Islamic faith. The majority said he was a sinful apostate who deserved damnation. Occasionally, during non-Muslim religious holidays and events, Kuwaiti religious clerics warn ordinary Muslims that the celebration of such events is prohibited by religion (U.S. Department of State, 2021). The reactions of some conservatives against free speech and expression can instill fear in those thinking of expressing anti-religious opinions. For example, in a survey of 1,050 Kuwaitis, Gengler et al. (2021) found that specific dimensions of religiosity positively predicted the endorsement of honor violence perpetrated by men against female relatives in cases of adultery. Half of the respondents favored such violence, a third favored the introduction of laws to punish adulterers, and interestingly, women respondents had similar attitudes to men.

At the level of Kuwaiti institutions, the perceived repression emanating from national institutions can contribute to the fear of social isolation. Al-Kandari et al. (2021) referred to types of institutional-driven fear in which institutions of authority use the law to bolster a repressive culture that suppresses opinions critical of Islam. Kuwaiti law criminalizes the publication and broadcasting of content that denigrates Allah, Islam, or any of the prophets. The law allows ordinary citizens to press criminal charges against any person who defames Islam or harms public morality. Occasionally, the government has arrested, interrogated, and prosecuted social media activists for speaking out against Islam. In 2021, authorities arrested a Kuwaiti citizen for blasphemy because he criticized Islam and praised secularism in his tweets. Many local social media users expressed delight and applauded the authorities for the arrest, and encouraged the ministry to do the same against anyone who insults Islam (U.S. Department of State, 2021).

As the issue of women recruitment in the military is strongly influenced by a conservative Islamic viewpoint (Alkhaldi, 2021; Human Rights Watch, 2023), it is to be expected that individuals fear the isolation of others if they express opinions that are not compatible with the Islamic viewpoint on this issue. Therefore, the following hypothesis is posited:

H2: The FSI will negatively predict opinion on the military recruitment of women in Kuwait.

#### 1.3.3 Internally and externally driven religious fears and opinion expression

In matters concerning religion, Muslims may fear not only social isolation but also direct divine retribution for expressing opinions that are not based on religion. Expressing opinions contrary to Islam can make one feel sinful and guilty if one believes it will lead to God's wrath and punishment in one's lifetime or the afterlife. In a study, Wyatt et al. (1996) surveyed causes of opinion stifling among Americans, Israeli Jews, and Israeli Arabs. They found that the Israeli Arabs, more than the others, suppressed the expression of their views on issues that conflicted with their religious principles. The present study adopts the concept of “internally-driven religious fear,” which corresponds to “repercussions for expressing opinions that violate religious dogma” (Al-Kandari et al., 2021, p. 315).

In contrast to religious fear based on internal guilt, the present study introduces an “externally driven religious fear” operationalized as the repercussions for expressing an opinion that may be deemed baseless by a religious community. The consequences of this fear include social punishment by others who would shame one for expressing views that contradict religious teachings. These punishments include castigation, social disapproval, and being labeled a heretic. This fear can be a stronger predictor of suppression of opinion than an internal fear because the latter is based on self-evaluation and a discrepancy between one's opinion and the teachings of the religion to which one belongs. On the other hand, external religious fears result from knowing that the public knows that one's opinion does not align with religious teachings and that one will be judged and possibly punished by others. Accordingly, the following is explored:

H3: An external religious fear is more likely to negatively predict the expression of opinion about the military recruitment of women in Kuwait than an internal religious fear.

#### 1.3.4 Religiosity and opinion congruency

Scheufele and Moy (2000) refer to SOS as a “theory of social perception” (p. 6), as perceptions of one's surroundings determine decisions about speaking up or remaining silent. According to Laluddin (2016, p. 20), “The search for an outside enemy or exaggeration of the presumed danger the enemy force may not actually pose not only maintains a group's structure, but it also strengthens it against the danger caused by relaxation of energies or internal dissension.” To maintain the status quo in the Arab public opinion, religious authorities have long portrayed Westernization and globalization as tools of hostile outsiders who target and seek to harm Islam and the Islamic way of life. The conservative religious community emphasizes Western support for women's rights in Muslim societies to get people to adhere to conservative religious interpretations of women's issues (Tripp, 2006).

Kuwait's culture resists change and restricts the flow of new ideas (Hofstede, 2022; Nydell, 2018). This culture can make people feel that religion is an obstacle to expressing critical opinions toward the religious community (Dashti et al., 2015). Occasionally, Islamist members of parliament pressure the Kuwaiti government to censor liberal voices that challenge prevailing conservative opinions. In recent years, Islamist members have conducted a parliamentary interpellation against a Kuwaiti Minister of Information and then removed him from office by a majority vote in parliament. The opposition claimed that the minister had allowed the exhibition of secular and anti-Islamic books at the Kuwait International Book Fair (Alqahtani, 2020). Some conservatives have pressured the government to ban the media from disseminating information about or symbolically supporting non-Muslim events. In recent years, Kuwaiti authorities removed a Christmas tree and a Roman statue from a shopping mall after opposition from conservatives grew on social media. The detractors claimed that such displays challenged Islamic norms (U.S. Department of State, 2021). These cases show that maintaining the status quo and upholding prevailing religious public opinion can influence individuals' expression of their views.

Regarding the SOS concept of opinion congruence about religion, Chaudhry and Gruzd (2020) argued that a negative personal opinion about a religion or religious belief causes individuals to silence their views on topics with religious overtones in public. About the topic of the satirical religious cartoons of the Prophet Muhammad published by the French magazine Charlie Hebdo, Fladmoe and Steen-Johnsen (2017) found that individuals silenced their opinions when they perceived their views to be incongruent with the dominant social positions on the religious issue. Accordingly:

H4: The perception that personal opinion congruency with that of the public will positively predict a person's opinion about Kuwait's military recruitment of women.

#### 1.3.5 Religious media uses and gratifications

This study relates SOS to U&G because people depend on media to receive cues about public opinion distribution on an issue (Al-Kandari et al., 2022). Earlier theories of mass communication assumed that the audience has only a limited influence on the effects of the media, positing that audiences passively and unconsciously absorb media messages and are influenced by their direct effects, whether they want to be or not. In contrast, the Uses and Gratification perspective views audiences as active and instrumental actors who extract the most rewarding psychological and social benefits from the media. The instrumentalization of audiences supposedly results in the filtering out and filtering messages that regulate and condition the media's influence on people. From this point of view, the mass media are seen as neutral, objective, and ineffective. They can only convey media messages to an audience whose use and participation determine their effects (Palmgreen et al., 1985).

This study integrates SOS and U&G to provide two alternative frameworks that may influence opinion expressions differently regarding a public opinion issue. SOS refers to the social influence on individuals' opinion expression while U&G is an audience-centered perspective that looks at how individuals have the ultimate control over their use of media, which indirectly influences their ways of opinion expression. For example, previous research suggests that people who use media mainly for information and more likely to feel they are informed about issues. This makes them more expressive of their opinions about different issues (e.g., Al-Kandari et al., 2022).

The early wave of research linking U&G to religion aimed to identify the motivations behind people's use of religious media. It states that people use religious media for entertainment, information and value-oriented purposes (Drumheller, 2005), passing time, learning and self-enhancement (Ratcliff et al., 2017), and ministering, spiritual enlightenment and information seeking (Brubaker and Haigh, 2017). The second wave explored factors that predict religious media usage, such as dissatisfaction with secular media (Abelman, 1987), one's religious denomination and level of faith (Woods et al., 2016), degrees of religiosity (Brubaker and Haigh, 2017), and religious maturity (Retpitasari and Oktavia, 2020). The final wave linked U&G to media engagement activities, such as the frequency of logging into Facebook for intense usage (Brubaker and Haigh, 2017) and the extent of online activity participation (Bentley, 2012).

Despite the value of these endeavors, previous research largely limits religious media to serving personal psychological motives and needs, except Brubaker and Haigh (2017), who found that Facebook faith content served the social function of ministering. This research reinforced criticisms previously leveled at early applications of U&G to secular media that the approach neglects the social roots of media usage (Cooper, 1997; Reimer, 1997). It also failed to link religious U&G to macro-social and political effects. Finally, it predominantly reflected a Christian perspective on religious media use.

Even though people of different faiths can obtain many similar religious satisfactions from the media, differences arise due to other religions' social, political, and ideological functions. Ayatollahy (2008) discusses the fact that Christianity and Buddhism focus more on the personal morality of the individual, while Islam and Judaism influence the lives of their followers through their religious jurisprudence; “Islam does not concern itself only with the personal relationship between man and God but also with the relationship between man and society” (Ayatollahy, 2008, p. 36). He maintains that “Islam emphasizes that the relationship between man and God is contingent on the fulfillment of the believer's social duties” (p. 36). Shahba and Hammam (2005) also argue, “(f)or the Islamic tradition there is no sharp division between the spiritual and the material worlds. For Muslims, the concepts of din (religion) and Dunya (the world) are interwoven” (p. 3).

The present study is based on the U&G study by Al-Kandari (2011) on Muslims' television usage, which divides the motives for using religious media into “individual” and “social.” The “individual” use is operationalized to reflect a personal usage that connects a person with Allah, acquires religious knowledge and information, comes closer to one's religion, and perceives the prophets as role models. On the other hand, “social” use connects a person to society. It refers to using religious media to discuss faith with others, preach, and defend the religion against critics.

Examining this categorization in the context of Samuel Huntington's notion of the Clash of Civilizations suggests that future wars and international tensions will be between Muslims and Westerners due to cultural differences. Al-Kandari (2011), for example, found that “individual” use predicted clash negatively, while “social” use predicted it positively. To explain the results, the study posited that “individual” use reflects a peaceful and spiritual person who aligns themselves with faith toward virtuous inner goals. Such a person avoids politically controversial and divisive religious judgments that divide others into “Us” vs. “Them.” Conversely, “social” use consists of religiously committed users who preach religion and defend it against secular attacks. They are examples of Noelle-Neumann's “hardcore”—ideologically oriented people who see religion as the solution to life's ills. In this context, the following is to be expected:

H5: The “social” use of religious media will more positively predict the expression of opinion about the military recruitment of women in Kuwait than the “individual” use of religious media.

#### 1.3.6 Religiosity and online opinion expression

Online SOS research in the context of opinion expression has led to contradictory results. However, the consequences can be explained theoretically from the “liberating” and “silencing” perspectives (Park, 2015). The “liberating” perspective states that online media liberates people from concerns about social isolation while undermining the influence of the climate of opinion on the judgment of expression. Research has found that the influence of the climate of opinion on expression is weaker online (Ho and McLeod, 2008) and that online media can mitigate and reduce conflict and create a diverse public climate of opinion (Schulz and Roessler, 2012). The diversity of opinion enables individuals to seek like-minded people to identify with and share opinions, which reduces the fear of being alone (Schulz and Roessler, 2012). Online “echo chambers” and “filter bubbles” (Pariser, 2011) allow individuals to select the people with whom they feel safe to share opinions. On the web, the existence of dominant majorities with differing views is masked because “algorithms inadvertently amplify ideological segregation by automatically recommending content an individual is likely to agree with” (Flaxman et al., 2016, p. 299). Concealing one's identity on web applications can increase one's sense of security and safety, which contributes to expressing unpopular opinions (Matthes, 2013). In contrast to offline contexts, where verbal and non-verbal communication make up interpersonal speech and communication, the absence of communicative cues online boosts self-confidence and removes the burden of public judgment (Ho and McLeod, 2008).

However, the “silencing” perspective suggests that an online environment influences the expression of opinions in the same way as offline environments. Many people transfer their social circles from the offline world to the online context, making them cautious when expressing socially offensive opinions (Pang et al., 2016). Moreover, through the intensive use of social media, people learn which statements lead to the loss of followers, which equates to a climate of opinion (Al-Sumait et al., 2021). People try to hide their political leanings online or avoid being critical of widely shared topics to prevent social isolation from people who “unfollow” them or online groups they dislike (Williams and Nida, 2011).

Research on the expression of opinion in Kuwait also provides inconclusive results. It shows that fears related to public opinion predict offline and, to a lesser extent, online expression (Al-Kandari et al., 2022) and presence (Moharrak et al., 2025). One study found that fear of legal persecution and fear of being ridiculed by others have similar effects on the suppression of certain opinions, both online and offline. Similarly, public opinion on platform X suppressed expression in both contexts (Al-Kandari et al., 2021). Another study found that a broader religious attitude toward an issue influenced opinion avoidance offline but not online (Al-Sumait et al., 2021). A study on the inclusion of women in the Kuwaiti police found that a broader religious attitude leads to opinion suppression offline and online. At the same time, personal characteristics such as outspokenness augmented opinion expression online and offline (Al-Kandari et al., 2021). Given the lack of solid confirmation of the influence of offline and online contexts on opinion expression in Kuwait, the first research question is posed:

RQ1: What are the factors (theoretical SOS components, religious predisposition, and religious media usage) that best predict the offline and online expression of opinion about women military recruitment in Kuwait?

## 2 Method

### 2.1 Population and sample

The population of this study was university students at Gulf University for Science and Technology, Kuwait. The number of students at the university is estimated by 5,000. A self-administered questionnaire that took about 15 min was filled out by a sample of 652 students who received extra credit for their contribution. To secure responses from students of diverse disciplines, the questionnaire was completed by students enrolled in general introductory courses in social studies, cultural studies, language, math, and the natural sciences. The questionnaire was originally written in English and then translated into Arabic to mitigate fluency in English from being a confounding variable in the research design. A university student sample suits this study's purpose as the demographic frequently accesses online media. Hence, they are more exposed to new ideas representing various ideologies and perspectives on politics, society, and life (Hasanen et al., 2014). Furthermore, they are more likely to be open to new trends than older Kuwaitis, who tend to be more conservative (Abdulrahim et al., 2009).

### 2.2 Dependent variables

This study consisted of two criterion variables: online and offline willingness to express an opinion. Both were introduced to respondents as a hypothetical scenario reflecting an incongruent opinion climate setting, adopted from Willnat et al. (2002). The scenario was constructed to reflect six characteristics to validly measure speaking out (Scheufele and Moy, 2000); they are: “cross-national differences, public exposure, anonymous public, size of the public, survey data, and moral loading” (p. 15). “Cross-national difference” refers to the fact that about 70% of the population of Kuwait are non-Kuwaiti citizens, suggesting a diverse public holding different outlooks. Regarding “public exposure,” Kuwaitis can view hundreds of satellite television channels (Abdulrahim et al., 2009), and the Digital Global Report (2022) indicates that internet penetration is around 99% of the total population, while social media penetration is around 93%. Those data suggest a great exposure to issues of public affairs. As for the “anonymous public,” the hypothetical scenarios this study presented to respondents asked them to imagine themselves in a gathering that included unknown people. Regarding the “size of the public,” the question did not indicate a specific number of individuals at the gathering, but one could infer that a typical gathering comprises ten to twenty individuals in a Kuwaiti cultural context. As for the “survey data” and “moral loading,” this study is based on a questionnaire connecting an issue about women to religion. As previously discussed, women's issues can fall under the umbrella of moral problems in Arab and Muslim cultures.

Respondents were given a hypothetical scenario to measure their willingness to express an opinion offline, including people they did not know who expressed contradictory views. Then, a question followed: their willingness to express an opinion and consider the situation. The item read: “Imagine you are in a social gathering and do not know most people there. A group of people start discussing the issue of female recruitment as officers and soldiers in the Kuwaiti military. How likely is it that you will express your opinion if the views of most of the people in the gathering are different from your own?” The wording was adapted for the second scenario in the online context to reflect an online scenario. It read, “Now imagine that the issue of female recruitment as officers and soldiers in the Kuwaiti military is being discussed or being posted about on X platform. How likely will you express your opinion about the issue if most people on X platform's views differ from yours?” For both of the previous scenarios, respondents expressed their willingness to express their opinions utilizing a five-point scale ranging from “Extremely Likely,” coded as 5, to “Extremely Unlikely,” coded as 1.

### 2.3 Predictor variables

This study included four main sets of predictor variables. Religious and socially related predictors included five variables. All of them were constructed using indices of four items. Fear of social isolation was adopted from Ho and McLeod (2008), internal religious fear from Al-Kandari et al. (2021), and religiosity and religious rituals from Krauss et al. (2007). The external religious fear was constructed from items reflecting the fear of communal reprisals. For all predictors, except for religious rituals, respondents expressed their attitudes to statements using a five-point Likert scale ranging from “Strongly Agree,” coded as 5, to “Strongly Disagree,” coded as 1. Religious rituals were assessed using an ordinal five-point scale indicating a frequency for performing various religious rituals ranging from “Always,” 5, to “Never,” 1. Table 1 includes item wordings and Cronbach alpha reliability scores for each variable in the Confirmatory Rotated Factor Analysis.

**Table 1:** Confirmatory rotated factors analysis of indices of the predictor variables.

	**Factors**						
	**1**	**2**	**3**	**4**	**5**	**6**	**7**
**Factor 1: personal use of religious media (*****M*** = **4.40**, ***SD*** = **0.62)**							
I use religious media content to increase my religious knowledge	**0.800**	0.203	0.010	−0.029	0.196	0.255	0.110
I use religious media content to reinforce my connection with my religion	**0.762**	0.196	0.023	−0.027	0.244	0.221	0.184
I use religious media content to add to my religious information	**0.759**	0.206	−0.033	−0.022	0.142	0.304	0.099
I use religious media content to get closer to Allah (God)	**0.746**	−0.007	−0.128	0.062	0.121	0.244	0.046
I use religious media content to perceive the prophets and messengers of Allah as role models	**0.603**	0.141	0.053	−0.001	0.226	0.076	0.170
**Factor 2: internally-driven religious fear (*****M*** = **4.21**, ***SD*** = **0.91)**							
I fear the punishment of Allah If I express an opinion that differs from the teachings of religion	0.187	**0.839**	−0.015	0.079	0.169	0.052	0.028
I think I will be guilty if I express an opinion that disagrees with Allah's guidelines	0.110	**0.820**	−0.028	0.152	0.151	0.054	0.061
I will feel sinful if I express and opinion disaccords with Allah's instructions	0.165	**0.811**	0.021	0.037	0.077	0.093	0.070
I feel afraid of expressing an opinion that disagrees with my religious instructions	0.101	**0.806**	0.059	0.195	0.136	0.155	0.031
**Factor 3: fear of social isolation (*****M*** = **2.39**, ***SD*** = **0.1.01)**							
I feel worried if nobody wants to be around me because of my opinion	−0.003	0.057	**0.853**	0.147	0.011	0.057	−0.028
I feel uncomfortable if I disagree with other people	−0.046	−0.015	**0.845**	0.203	−0.070	0.031	0.046
I avoid telling other people what I think when there is a risk that they will avoid me if they knew my opinion	0.038	−0.041	**0.793**	0.225	0.023	0.065	−0.057
I worry about being isolated if people disagree with me	−0.062	0.023	**0.765**	0.327	−0.032	0.034	0.066
**Factor 4: externally-driven religious fear (*****M*** = **2.89**, ***SD*** = **1.09)**							
I will keep my opinion to myself if I feel others may judge my opinion as disaccording with the religion	0.023	0.025	0.214	**0.857**	0.019	0.040	−0.021
Sometimes I feel afraid that expressing my own opinion, if it disagrees with religious instructions, may make people form a negative impression about me	−0.032	0.162	0.192	**0.813**	0.137	0.074	−0.008
Sometimes I avoid expressing my personal opinion if it would make others categorize me as secular or liberal	0.057	0.099	0.244	**0.780**	0.056	0.074	0.050
I avoid expressing my opinion if I feel that others may label me as a religiously non-believer	−0.044	0.193	0.260	**0.777**	0.057	0.059	0.081
**Factor 5: religiosity (*****M*** = **4.20**, ***SD*** = **0.72)**							
I avoid doing something if I am unsure of its religious implications	0.151	0.137	−0.051	0.085	**0.820**	0.118	0.145
I make effort to obey rules/advice of my religion in my daily life	0.173	0.162	0.017	0.034	**0.788**	0.186	0.172
I make effort to always follow Islamic instructions in my life	0.207	0.114	0.045	0.079	**0.730**	0.072	0.108
I make efforts to understand the demands/obligations/teachings of my religion	0.285	0.150	−0.104	0.072	**0.712**	0.177	0.116
**Factor 6: social use of religious media (*****M*** = **4.04**, ***SD*** = **0.69)**							
I use religious media content to obtain information I can use in discussions about religion with others	0.204	0.011	0.070	0.084	0.095	**0.772**	0.133
I use religious media content to tell others what to do when it comes to religion	0.210	0.125	0.084	0.093	0.163	**0.710**	0.110
I use religious media content to be able to answer the religious questions others might ask	0.160	0.011	0.036	0.023	−0.005	**0.696**	0.230
I use religious media content to defend my religion when I have to	0.310	0.167	0.046	0.005	0.251	**0.553**	−0.077
I use religious media content to defend my religion against those who criticize it	0.276	0.258	−0.027	0.101	0.258	**0.501**	0.065
**Factor 7: religious prayers and rituals (*****M*** = **14.2**, ***SD*** = **3.26)**							
I conduct Qyam Aleel (salat) prayers at least once a week	0.062	0.056	0.033	0.123	0.190	0.266	**0.719**
I make an ongoing effort to increase the frequency of my non-obligatory (nafil) prayers	−0.001	0.087	0.137	−0.028	0.060	0.283	**0.718**
I practice (salat) religious prayers on time	0.196	0.102	0.035	0.027	0.307	−0.014	**0.665**
Eigenvalue	7.909	4.373	2.314	1.732	1.433	1.320	1.119
Pct. of variance explained	26.36	14.57	7.712	5.774	4.778	4.400	3.729
Alpha (reliability scores)	0.86	0.88	0.87	0.87	0.85	0.80	0.70

Loadings over 0.50 appear in bold.

The second predictor included religious media variables. Individual and social use of religious media was adopted from Al-Kandari (2011). Each contained an index of five survey items. Respondents indicated their attitudes toward the statements employing a five-point Likert scale of “Strongly Agree,” 5, to “Strongly Disagree,” 1. The frequency of exposure to religious media per day was another predictor. To report their answers, respondents choose from the following options: “Always,” coded as 5, “Often,” 4, “Sometimes,” 3, “Rarely,” 2, and “Never,” 1.

There were also items about opinion congruency. Respondents indicated levels of correspondence between their own opinions about female military recruitment and those of (1) the majority of individuals in Kuwaiti society (current society's opinion congruency with that of the respondent), (2) the majority of individuals in Kuwaiti society in the future (future society's opinion congruency with that of the respondent), (3) the media coverage of female military recruitment (media coverage congruency with the perception of the respondent), and (4) the majority of people on X platform (X platform opinion congruency with that of the respondent). For all items except media coverage, respondents used a bipolar scale of 1–10, where 1 reflected “Very dissimilar to mine” at 1 end and 10 as “Very similar to mine” at the second end. Respondents reported their evaluation of media coverage using a bipolar scale of 1–10, where one reflected “Very negative” and 10 “Very positive.”

X platform variables were another set of predictors. For the frequency of daily X platform use, respondents indicated their average usage employing a five-point scale ranging from “Always,” 5, to “Never,” 1. Respondents indicated whether they included their real names and personal pictures on their X platform account profiles for the X platform profile variable. The nominal options of inclusion were: “Yes, both (my real name and personal image),” coded as 4, “Only my personal image,” 3, “Only my real name,” 2, “I do not post either,” 1. Lastly, gender was the only demographic item in the study. Respondents indicated themselves as a “Male,” 1, or “Female,” 2.

A Confirmatory Rotated Factor Analysis was conducted using the Principal Components for extraction based on an eigenvalue value of at least 1 and Varimax rotation for all previous predictors made of indices. The procedure was meant to group items composing each factor (predictor variable). The factor analysis extracted seven factors, accounting for 67% of the total explained variance (Table 1).

### 2.4 Profile of the respondents

The sample comprised 652 respondents, 381 (58.5%) male and 271 (41.5%) female. 81 (12.4%) reported they did not use the X platform, 104 (16%) used it rarely, 208 (31.9%) sometimes, 137 (24%) often, and 122 (18.7%) always. 82 (12.6%) said they included neither their real names nor personal images in their account profile, 125 (19.2%) included only their real name, 32 (4.9%) only a personal image, and 323 (49.4%) both. The respondents were more willing to express their opinions offline (*M* = 3.38/5, *SD* = 1.37) than online (*M* = 2.65/5, *SD* = 1.50). Concerning the respondents' perceptions of opinion congruency, the statistics were as follows: opinion congruency with the society (*M* = 4.24/10, *SD* = 2.37), with the future (*M* = 5.62/10, *SD* = 2.65), with the media (*M* = 5.56/10, *SD* = 2.53), and on X platform (*M* = 4.89/10, *SD* = 2.54). The data indicates that the issue of women working in the military divides society and hence is suitable for a SOS study where opinions are expressed or silenced. According to Noelle-Neumann (1991), “The more clear-cut the majority and the minority are in the climate of opinion, the more it may be assumed that this will influence the willingness to speak out or keep silent in public” (p. 262).

## 3 Results

### 3.1 Statistical design and analysis

To explore the research question and hypotheses, two Multiple Linear Regression (MLR) tests were conducted, one for exploring factors predicting an offline expression of opinion and another for the predictors of an online expression of opinion. Predictor variables were entered in the independent box of the MLR test in the Statistical Package for the Social Sciences (SPSS). Gender was entered in the first level, religion-social-related predictors in the second, and media-related predictors in the last. The logic for this order was to control the effects of gender and to focus attention on the influence of the second and third-level predictor variables. For the second level, religious and social-related predictors were embedded. Those predictors were the religious predispositions having a significant influence on the attitudes of Muslims regarding different human phenomena and the different types of fears (fear of social isolation and internally and externally driven religious fears). The last level of MLR included media-related predictors (frequency of using religious media, individual and social religious media use, opinion congruency indicators, and X platform use variables).

Finally, opinion expression in offline and online settings as criterion variables were entered in the dependent variable box of the MLR separately, one at a time.

### 3.2 Offline opinion expression

The regression for the first MLR, testing the prediction of opinion expression in an offline setting, was not significant at the first level of analysis, which tested only gender [*R*^2^ change = 0.001, *F* change_(619, 1)_ = 0.184, *p* = 0.668]. At the second level, which considered gender and religious and social-related predictors, the MLR was significant [*R*^2^ change = 0.056, *F* change_(614, 5)_ = 7.255, *p* = 0.001]. Of all religious and social-related factors, fear of social isolation (β = −0.116, *p* = 0.014) and external religious fear (β = −0.128, *p* = 0.009) were negative predictors of offline opinion expression. Also, religiosity positively predicted an offline opinion expression (β = 0.101, *p* = 0.026). Finally, when entering media-related predictors in the last block, the MLR was significant [*R*^2^ change = 0.013, *F* change_(608, 6)_ = 1.401, *p* = 0.001]. At this level, religious and social-related predictors, fear of social isolation (β = −0.106, *p* = 0.027), and the external religious fear (β = −0.135, *p* = 0.006) remained significant predictors of an offline opinion expression, while religiosity lost its significance (β = 0.071, *p* = 0.148). The outcomes suggested that the more people feared social isolation from others and communal religious judgment, the more they were willing to suppress their opinions (Table 2).

**Table 2:** Multiple regression analyses of the predictors of offline and online opinion expression.

	**Offline opinion expression**			**Online opinion expression**		
	β	***Std error***	***p***	β	***Std error***	***p***
**Demographic**						
Gender	−0.061	0.114	−0.022	0.054	0.141	0.018
**Religious & social-related factors**						
Fear of social Isolation	−0.134	0.060	−0.106*	0.027	0.071	0.019
Internal religious Fear	0.041	0.070	0.027	−0.045	0.083	−0.027
External religious Fear	−0.168	0.062	−0.135^**^	−0.138	0.072	−0.101
Religiosity	0.131	0.094	0.068	0.261	0.114	0.124^***^
Religious rituals & prayers	0.014	0.019	0.033	−0.042	0.023	−0.090
**Media-related factors**						
Congruency in society	0.009	0.026	0.015	0.009	0.032	0.014
Congruency in future	−0.018	0.023	−0.035	−0.046	0.028	−0.83
Congruency in media	0.024	0.023	0.044	−0.013	0.027	−0.022
Congruency in *X* platform	–	–	–	0.016	0.030	0.027
Frequency of using religious media	−0.104	0.054	−0.086	0.019	0.065	0.014
Personal religious media use	0.130	0.119	0.058	−0.190	0.139	−0.079
Social religious media use	0.128	0.102	0.065	0.307	0.118	0.143^**^
Daily use of *X*	–	–	–	0.254	0.065	0.174^*^
X's user profile	–	–	–	−0.025	0.057	−0.020
**Model significant statistics**						
R^2^	0.069			0.062		
Adjusted *R*^2^	0.051			0.035		
*R*^2^ change	0.013			0.045		
F change	1.401			2.824		

^*^p < 0.05, ^**^p < 0.01, ^***^p < 0.001.

### 3.3 Online opinion expression

In the second MLR, examining the predictors of an opinion expression online (X platform), was not significant at the first level [*R*^2^ change = 0.000, *F* change_(541, 1)_ = 0.021, *p* = 0.886], nor the second level [*R*^2^ change = 0.017, *F* change_(536, 5)_ = 1.809, *p* = 0.109]. However, when considering the media-related predictors in the last block, the MLR was significant [*R*^2^ change = 0.045, *F* change_(527, 9)_ = 2.624, *p* = 0.003]. At this level, religiosity (β = 0.124, *p* = 0.022), the social-religious use of media (β = 0.143, *p* = 0.010), and the daily average use of X (β = 0.174, *p* = 0.001) were all positive predictors of online opinion expression. The more people felt they were religious, used the media for social and religious reasons, and used X daily, the more they were willing to express their opinions online (X platform) (Table 2).

### 3.4 Results of the hypotheses and research question

In response to the hypotheses and research question, the results that the first hypothesis, which stated that, “Religiously committed individuals are more likely to express their opinions about Kuwait's recruitment of women into the military,” was partially confirmed. Religiosity was a positive predictor of opinion expression only in online settings. Individuals who felt more religious were more likely to express their opinions about the issue online. As for the second hypothesis that stated, “The FSI will negatively predict opinion on the military recruitment of women in Kuwait,” it was also partially confirmed. FSI was a negative predictor of opinion expression only offline. Individuals who feared social isolation were less likely to express their opinion about the issue. The third hypothesis, which stated, “An external religious fear is more likely to negatively predict the expression of opinion about the military recruitment of women in Kuwait than an internal religious fear,” was partially confirmed too. The external religious fear was a negative predictor of opinion expression in offline settings. Individuals who feared the others tried to silence their opinions about the issue. The fourth hypothesis stated, “The perception that personal opinion congruency with that of the public will positively predict a person's opinion about Kuwait's military recruitment of women”. This hypothesis was rejected for offline and online settings. The fifth hypothesis stated, “The “social” use of religious media will more positively predict the expression of opinion about the military recruitment of women in Kuwait than the “individual” use of religious media”. The hypothesis was partially confirmed. Individuals who used religious media for “social” use were more outspoken about their opinions in online settings. Finally, regarding the research question, which asked about the factors that best predicted opinion expressions about the issue in offline and online setting, the results indicated that the frequency of use of X platform followed by the “social” use of religious media were the strongest predictors of opinion expression.

## 4 Discussion

This study examines the influence of religion on opinion expression. The first section discusses offline and online opinion predictors on a publicly divisive and moral issue: recruiting women into the military in Kuwait. The second section concludes by examining the influence of the online atmosphere on opinion expression in Arab culture.

### 4.1 Offline opinion expression

This study found that fear of social isolation predicts opinion avoidance in offline contexts. This result supports Noelle-Neumann's (1977, 1991) theoretical hypothesis that fear of social isolation is the main reason for not expressing a minority opinion. The results align with other SOS studies (Scheufele and Moy, 2000). This study also found that externally driven religious fear and fear of backlash from the religious community negatively predicted opinion expression. Although fear of social isolation and externally-driven religious fear negatively predicted opinion expression, the effect size for externally-driven religious fear was larger. This suggests that the fear of reprisals from the religious community has a stronger influence on the decision not to speak out than the fear of isolation. This could indicate that religion has a greater impact on people than cultural conventions and norms.

Similar to previous studies on SOS in Kuwait, internally driven fear (guilt-driven fear) did not predict avoidance of expressing an opinion. However, externally driven fear (shame-driven fear) predicted suppression of opinion. None of the predictors of religiosity predicted opinion expression in offline contexts. Similarly, none of the factors for public opinion congruence (congruence with society's views at present, congruence with society's views in the future, or perceptions of media coverage) predicted the expression of opinions offline.

Concerning the previous results, Noelle-Neumann (1977) spoke of public opinion being in a “liquid” and a “solid” state. She elaborated that (p. 145) “In societies…where social change is slow, no strenuous observation of the social environment is necessary to avoid isolation: the norms, expected and approved patterns of behavior, are known as well as the dominant opinions.” She also argued, “Where opinions are relatively definite and static…one has to express or act according to this opinion in public or run the risk of becoming isolated. In contrast, where opinions are in flux or disputed, the individual will try to find out which opinion he can express without becoming isolated” (Noelle-Neumann, 1977, p. 145). Noelle-Neumann's argument can explain why religiosity does not predict opinion expression.

Compared to Western, liberal, and democratic nations, public opinion in Kuwait is solid and static, and change is slow. The prevailing conservative and religious public opinion resists change and often restricts the flow of new ideas and perspectives (Hofstede, 2022; Nydell, 2018). In such a state, congruence of opinion factors does not matter to people as they are accustomed to the stagnancy and are aware of the limits of what is acceptable when discussing most topics. Accordingly, the results showed that the congruence of the opinion climate does not influence the willingness to express an opinion. In addition, the religious dominance of public opinion, where any defiance of religion is unacceptable, means that people were less affected by their religious inclinations to express themselves than in the United States, where the literature (Alkazemi, 2013; Croucher et al., 2014) suggests that religiosity predicts people's expression of opinion. The liquid state of public opinion in the United States promotes diversity of perspectives on issues, and people are encouraged to defend their opinions by any means. In Kuwait, where religion plays a greater role, religious predispositions may be less critical because public opinion suppresses other perspectives and ways of thinking. There is no need to defend and protect something already protected by the dominant culture.

### 4.2 Online opinion expression

In online contexts, the influence of fear of social isolation and externally driven religious fears do not play a role. People were not afraid of social ostracism or being labeled religious in their social circles. This suggests that online media provides free channels for expression where people do not feel pressured by their immediate culture or religious community to suppress their opinions. This discovery is consistent with the “liberation” perspective (Park, 2015). Previous research suggests that online media liberates people from pressure in offline contexts. The pressure of the opinion climate on congruence is weak online (Ho and McLeod, 2008) because people say what they believe regardless of what others think. Online media soften sharp opinion divisions (Schulz and Roessler, 2012) because people feel equal online egalitarianism and do not consider themselves a minority. Diversity of opinion, finding like-minded people (Schulz and Roessler, 2012), online “echo chambers” and “filter bubbles” (Pariser, 2011) reduce people's concern about being alone, which reduces the fear of social isolation and reduces the importance of the climate of opinion.

In contrast to offline contexts, where religiosity did not predict opinion expression, the predictor triggered opinion expression in online contexts. Again, the distinction between these “solid” and “liquid” states of public opinion is important (Noelle-Neumann, 1977). Online contexts are liquid enough to contain diverse views on topics where it is challenging for people to defend their views. “Social” use of religious media positively predicted opinion expression in online contexts, but not offline. The diversity of online opinions encourages different viewpoints, and people use religious media content to defend their religious beliefs.

The fact that “social” use of religious media rather than “individual” use of religious media positively predicted opinion expression is interesting because, in contrast to the American setting where the U&G perspective on religious media is centered on Christianity and online content is used for personal psychological motives (e.g., Brubaker and Haigh, 2017; Drumheller, 2005; Ratcliff et al., 2017), the current study found that social motives were more important. The U&G perspective has sometimes been criticized for ignoring these social roots. However, the “social” use of religious media predicts opinion expression in online contexts in Kuwait. The “social” use of religious media implies that people use it to connect with society, discuss their faith, preach about it, and defend it if necessary. It galvanizes people who are religiously committed, sometimes dogmatic, and find religious solutions to their everyday social problems and issues. They can be hardcore and/or ideologically motivated to express their views on the military recruitment of women. On the other hand, “individual” use of religious media refers to people who use it for personal, psychological, and spiritual reasons, such as getting closer to Allah and using information from religious media for their purposes. They are usually peaceful and avoid expressing controversial opinions to avoid dividing society (Al-Kandari, 2011).

Finally, frequent daily use of the X platform positively predicted opinion expression. The more people used the X platform, the more they wanted to express their opinions. Individuals who engage more in online environments develop an awareness of online norms of public expression and learn not to offend their communities so their followers do not vote them out. Thus, they are essentially “encultured” by the online context and become proficient in its nuances (Al-Sumait et al., 2021).

## 5 Conclusion

The fact that this study found that fear of social isolation and externally driven religious fear negatively predicted opinion expression in offline contexts suggests the influence of culture in offline contexts. The study also found that religious inclinations, how individuals use media for “social” religious motives, and daily use of X platforms positively predicted expression in online contexts. As these indicators are personal in nature, this suggests that culture has a greater effect in offline contexts and that personality and individual characteristics are influential in online contexts. The predictors for the offline opinion expression were all negative, and those for the online opinion expression were all positive. This means that the offline culture was restrictive, and the online culture was generally liberating. The results of SOS suggest that a rational perspective (Scheufele and Moy, 2000) on SOS needs to be emphasized when exploring opinion expression in online contexts. The results also indicate that the standpoint of conformity (Scheufele and Moy, 2000), which has dominated the SOS literature, will continue to be productive, mainly in offline contexts.

The results indicate that social media in collective Arab cultures can bolster people's individualistic tendencies and promote freedom of expression. Kuwaitis can find places to freely express their opinions online by avoiding their sometimes-repressive offline cultures. In the long run, online media can encourage a more expressive population. This can also lead society to transfer online opinions to offline contexts and enrich social debate.

Finally, like any other social science research, this study has limitations. It was conducted exclusively among university students in Kuwait, young people who are usually liberal and open to change (Abdulrahim et al., 2009) and generally willing to accept new progressive family roles for women (Hasanen et al., 2014). However, if this study had been carried out on the older, more traditionally inclined population, religious predispositions would probably have had a greater influence on the opinions expressed.

## Data Availability

The raw data supporting the conclusions of this article will be made available by the authors, without undue reservation.
